# Proximal femoral bionic nail (PFBN) versus InterTAN nail for intertrochanteric femoral fractures in elderly patients: a systematic review and meta-analysis

**DOI:** 10.1186/s12893-026-03943-8

**Published:** 2026-06-20

**Authors:** Hongqin Li, Yong Deng, Jiongnan Xu, Xianghua Xiong, Xiang Li, Tingxiao Zhao, Qifeng Ying, Jun Zhang

**Affiliations:** 1 Guizhou Medical University, Guiyang, Guizhou 550004 China; 2 The People’s Hospital of Chongqing Liangping District, Chongqing, 405200 China; 3 Zunyi Medical University, Zunyi, Guizhou 563000 China; 4 Department of Orthopedics, Zhejiang Provincial People’s Hospital Bijie Hospital (The First People’s Hospital of Bijie), 551700 Bijie, Guizhou,, China; 5 Center for Plastic & Reconstructive Surgery, Department of Orthopedics, Zhejiang Provincial People’s Hospital, Affiliated People’s Hospital, Hangzhou Medical College, Hangzhou, Zhejiang 310000 China; 6 Center of Osteoporosis, Zhejiang Provincial People’s Hospital, Hangzhou, Zhejiang 310000 China

**Keywords:** PFBN, InterTAN nail, Intertrochanteric femoral fracture, Intramedullary nail fixation, Meta-analysis

## Abstract

**Objective:**

To systematically assess the efficacy and safety of the proximal femoral bionic nail (PFBN) compared with the InterTAN nail in the management of intertrochanteric femoral fractures (IFFs) among elderly individuals.

**Methods:**

Eligibility criteria (inclusion and exclusion) were established in accordance with the PICOS framework. Relevant clinical studies were systematically retrieved from both Chinese (CNKI, VIP, and Wanfang Data) and English databases (PubMed, Embase, Web of Science, the Cochrane Library) with a search cutoff date of October 31, 2025. The Risk of Bias in Non-randomized Studies of Interventions (ROBINS-I) tool was employed to appraise methodological quality of the included literature. Meta-analyses of outcome measures, including intraoperative duration, intraoperative haemorrhage, length of hospital stay, postoperative weight-bearing duration, Harris Hip Score (HHS), and internal fixation-related complications, were conducted using RevMan 5.3 and Stata 15 software.

**Results:**

Six relevant studies were finally enrolled in the present analysis, consisting of 2 prospective cohort studies and 4 retrospective cohort studies, with a total of 683 participants. Among all cases, 334 patients were allocated to the PFBN group and the remaining 349 cases received InterTAN nail fixation. Meta-analysis outcomes demonstrated no significant inter-group differences in intraoperative blood loss (*P* = 0.17), postoperative weight-bearing time (*P* = 0.47) and Harris Hip Score (HHS) (*P* = 0.11). When compared with the InterTAN nail system, the PFBN group presented a shorter hospital stay (*P* = 0.04) and a decreased rate of internal fixation-related complications (*P* = 0.01), whereas the PFBN group had a longer operative time (*P* = 0.03). Sensitivity analysis further confirmed the good overall stability and reliability of the pooled outcomes in this meta-analysis.

**Conclusion:**

Based on the limited evidence from current non-randomized studies, PFBN exhibits potential benefits in shortening hospital duration and reducing the risk of internal fixation-related complications among elderly patients with intertrochanteric femoral fractures. Meanwhile, this implant yields equivalent effects to the InterTAN nail with regard to functional recovery. Given the observational design of all included trials and substantial heterogeneity in several outcomes, these findings warrant cautious interpretation.

**Supplementary Information:**

The online version contains supplementary material available at https://doi.org/10.1186/s12893-026-03943-8.

## Introduction

With the accelerating global aging demographic trend, the prevalence of osteoporosis in the elderly population is increasing significantly, which directly leads to a yearly rise in the incidence of hip fractures [1–8]. As one of the predominant types of hip fractures, intertrochanteric femoral fractures (IFFs) in the elderly not only severely impair patients’ health-related quality of life [9, 10] but also substantially increase the burden on the healthcare system, representing a major clinical challenge in orthopaedic practice [1, 3, 11–16].

In the surgical management of elderly IFFs, treatment outcomes depend heavily on the selection of internal fixation devices. Currently, clinically applied internal fixation systems are mainly classified into extramedullary and intramedullary fixation systems [4, 6, 17]. Extramedullary fixation is represented by the AO angular compression plate and dynamic hip screw (DHS); intramedullary fixation includes the Gamma nail, InterTAN nail, and proximal femoral nail anti-rotation (PFNA) [15, 18–21]. Compared with extramedullary fixation systems, intramedullary devices offer superior biomechanical stability, as the implant is placed within the femoral medullary canal, resulting in a shorter lever arm and optimized stress distribution. Additionally, these systems have clinical advantages such as minimal surgical trauma, reduced intraoperative duration, and less blood loss, making them more suitable for elderly patients with osteoporosis and unstable fracture patterns [5, 17, 21, 22]. However, with the widespread clinical adoption of intramedullary nails, associated complications have gradually become apparent, including screw cut-out, implant loosening or fracture, and postoperative femoral varus deformity [22–24]. These complications not only hinder fracture healing and treatment efficacy but also increase the risk of secondary surgery in affected patients, making intramedullary nail innovation a key research focus. Among these advancements, proximal femoral bionic nail (PFBN), a novel modified intramedullary fixation device developed in recent years, has been clinically validated to reduce the incidence of internal fixation failure in IFFs [19, 25, 26]. A previous meta-analysis [27] indicated that compared with PFNA, the PFBN provides enhanced stability and biomechanical advantages, enabling earlier weight bearing and accelerated fracture healing. It has gradually become a research hotspot in the field of internal fixation for elderly IFFs, attracting growing clinical and research interest [18–20].

As a new internal fixation system for the treatment of elderly IFFs, PFBN and the commonly used InterTAN nail both have unique clinical advantages. Derived from the PFNA [27], PFBN has been validated by multiple finite element analyses to have favorable mechanical properties, effectively reducing the risk of stress concentration, fracture, and loosening between screws [28–31]. Based on the biomechanical principle of the “N-triangle theory” of the proximal femur [27–29, 31–37], this technology innovatively incorporates an additional transverse screw crossing the femoral head screw. Through the rigid integration of this transverse screw with the nail holes and threaded structure of the intramedullary nail shaft, a stable triangular supportive mechanical system is formed [19, 20]. The dual-screw fixation mode can effectively resist rotational and shear forces, reducing the incidence of internal fixation failure (e.g., screw cut-out or backout) [19, 28, 31]. Meanwhile, the InterTAN nail is a well-established internal fixation system with favorable biomechanical performance and stable fixation, which lowers internal fixation-related complications [16, 38, 39]. It also shortens patients’ bed rest duration and reduces complications associated with prolonged immobilization, making it widely used in clinical practice [39].

Currently, most clinical studies comparing PFBN and the InterTAN nail are limited by small sample sizes, and their efficacy remains controversial. Therefore, a comprehensive and systematic meta-analysis of available evidence is needed to impartially and accurately compare the clinical outcomes of these two surgical methods in the treatment of elderly IFFs, to provide a robust evidence-based foundation for clinical practice decisions.

## Data and methods

Strict adherence to the PRISMA (Preferred Reporting Items for Systematic Reviews and Meta-Analyses) guidelines was maintained throughout this study, thereby safeguarding the transparency and replicability of the research outcomes. Furthermore, this meta-analysis was prospectively registered in PROSPERO and the assigned registration number is CRD420251153675.

### Eligibility criteria

The study selection criteria for literature screening were established based on the PICOS framework.

### Inclusion criteria

(1) Study population: Patients clinically diagnosed with intertrochanteric femoral fracture who were ≥ 65 years old; (2) Research design frameworks: randomized controlled trials (RCTs), retrospective studies and prospective cohort studies; (3) Interventions: Patients in the two cohorts were treated with PFBN or InterTAN nail, respectively; (4) Literature sources: Clinical studies comparing PFBN and InterTAN nail for the treatment of IFFs in elderly patients.

### Criteria for exclusion

(1) Duplicate publications; (2) Review articles, bibliometric studies, conference abstracts, and other study types that do not provide key outcome data; (3) Studies with unclear research design or substantial methodological flaws; (4) Studies with insufficient key data for extraction.

### Literature search strategy

Two independent investigators performed a comprehensive literature search across seven databases: PubMed, CNKI, VIP, Wanfang Data, Web of Science, the Cochrane Library, and Embase. A systematic combination of Medical Subject Headings (MeSH) terms and free-text keywords was employed to screen eligible studies published up to October 31, 2025 (as a paradigmatic example, the PubMed search strategy is detailed in Table 1). The indexed keywords included Intertrochanteric Femoral Fractures, Proximal Femoral Fracture, Femoral Fracture, Hip Fractures, Fractures, Intertrochanteric, Proximal Femoral Bionic nail and PFBN, along with their various permutations. All obtained references were imported into NoteExpress software for data management and duplicate removal.

**Table 1:** Search strategy of PubMed

Search	Query	Results
#1	Intertrochanteric Femoral Fractures[Mesh]	2906
#2	Fractures[Title/Abstract] OR Hip Fractures[Title/Abstract] OR Femoral	219551
	Fracture[Title/Abstract] OR Proximal Femoral Fracture[Title/Abstract] OR	
	Intertrochanteric[Title/Abstract] OR Intertrochanteric Femoral	
	Fractures[Title/Abstract]	
#3	#1 OR #2	219551
#4	Proximal Femoral Bionic Nail[Title/Abstract] OR PFBN[Title/Abstract]	28
#5	InterTAN Nail [Title/Abstract] OR InterTAN[Title/Abstract]	161
#6	#3 AND #4 AND #5	12

### Data extraction process

Data from eligible studies were extracted by two independent reviewers utilizing a pre-tested standardized data collection form. The following variables were systematically retrieved from the 6 included studies: author affiliations, publication year, study design, patient baseline characteristics, sample size, fracture classification system, follow-up period, operative duration, intraoperative haemorrhage, hospital length of stay, postoperative weight-bearing initiation, Harris Hip Score (HHS), and complications associated with internal fixation. For incomplete datasets, corresponding authors were contacted to provide supplementary information. Any discrepancies during data extraction were resolved by a third independent reviewer. For three-arm trials involving three comparative groups, only raw data from the PFBN and InterTAN arms were extracted, while data from the third arm were excluded from the present analysis. All data extraction procedures were independently performed by two investigators to avoid duplicate counting and inappropriate application of analytical units, thereby ensuring standardization and reliability of the subsequent statistical analyses.

### Cohorting method

For analytical purposes, patients in the included studies were stratified into two cohorts based on intervention type: the experimental cohort comprised patients treated with PFBN, while the control cohort included those managed with InterTAN nail fixation.

### Quality appraisal

Two independent researchers conducted a methodological quality appraisal of the included studies using the ROBINS-I tool (the Cochrane Risk of Bias Assessment Tool designed for non-randomized intervention studies). This appraisal addressed seven distinct domains of evaluation: confounding-linked bias, selection-related bias, bias in intervention classification, bias stemming from deviations from intended interventions, bias due to missing data, outcome assessment bias, and reporting-associated bias. For each domain, bias risk was categorized into five levels: low, moderate, serious, critical, and insufficient information. Any disagreements that arose during the quality evaluation process were resolved via consultation with a third independent researcher.

### Heterogeneity assessment

Between-study heterogeneity was evaluated using the χ² test and I² statistic. Significant heterogeneity was defined as a χ² test P-value < 0.10 and I² > 50%. Model selection followed these principles: a fixed-effects model was adopted if I² ≤ 50%, whereas a random-effects model was used otherwise. For the small number of studies (< 10) and substantial heterogeneity (I² > 50%), we critically appraised potential sources of heterogeneity. Further exploration of sources of heterogeneity was not feasible.

### Statistical analysis

RevMan 5.3 and Stata 15 software were employed for meta-analysis. Regarding binary categorical variables, effect sizes were determined based on study design: risk ratio (RR) for cohort studies and odds ratio (OR) for parallel controlled studies. In the case of continuous outcome measures, the weighted mean difference (WMD) was applied if outcome measures were reported with consistent assessment metrics across all included studies; otherwise, the standardized mean difference (SMD) was utilized for data pooling. All effect estimates were presented with 95% confidence intervals (95% CI), and statistical significance was set at a threshold of P < 0.05. Pooled effect sizes were visualized using forest plots, thereby offering a visual illustration of the meta-analysis findings.

## Results

### Study selection and basic characteristics

**Fig. 1:**
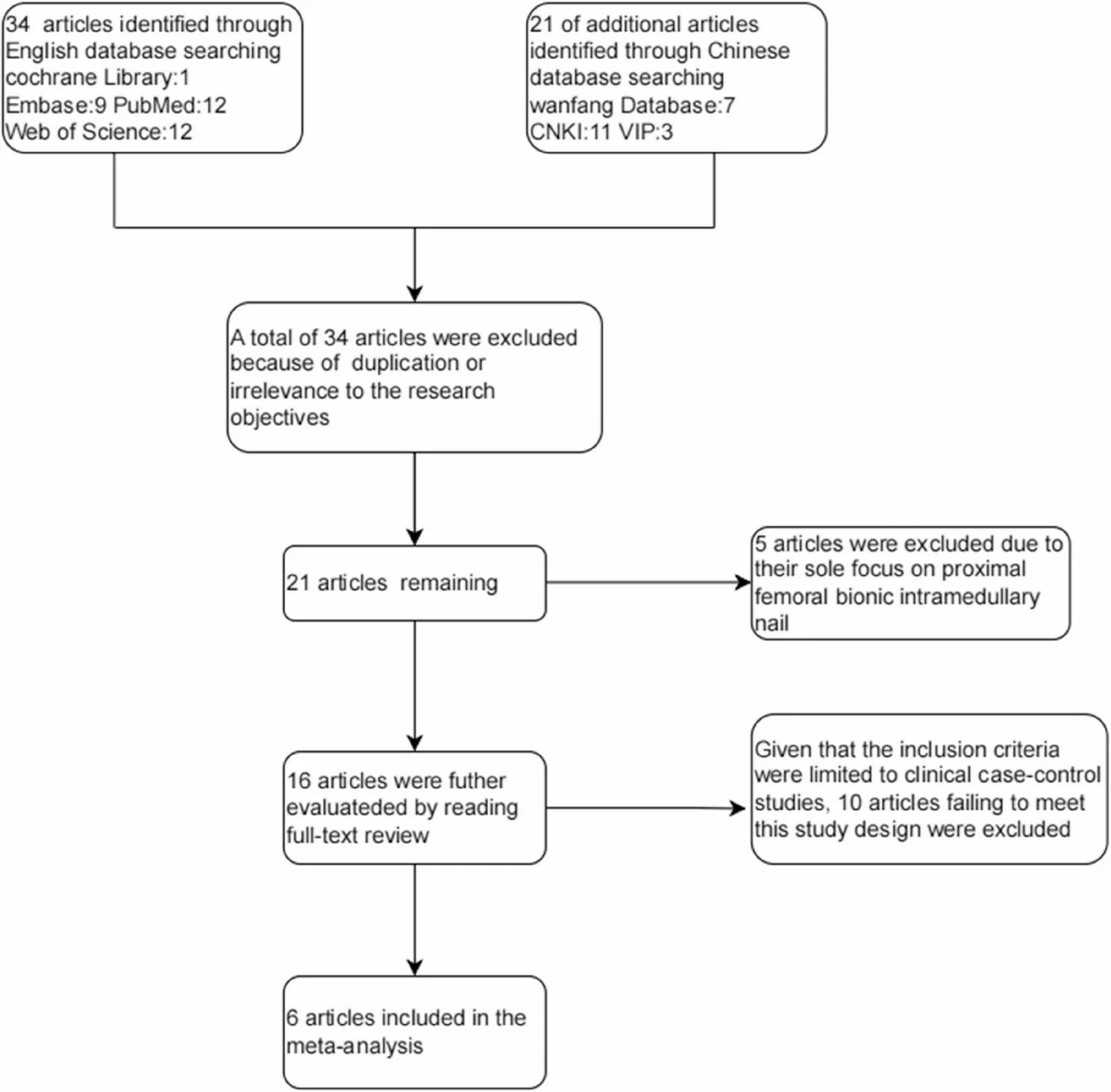
PRISMA flowchart of literature screening process in the meta-analysis

A total of 53 studies were retrieved following systematic database retrieval. After excluding duplicate or irrelevant studies, 20 studies were obtained following initial screening. Following further screening based on titles and abstracts, 16 studies were enrolled for full-text evaluation. Eventually, in strict adherence to the predefined study selection criteria, six studies [18, 35, 40–43] were included (Fig. 1). Among them, there were two prospective studies [41, 42] and four retrospective cohort studies [18, 35, 40, 43]; five were Chinese-language studies [18, 35, 40–42] and one was an English-language study [43]. All included studies, which were published between 2023 and 2025, collectively recruited a total of 683 patients. Among these participants, 334 patients in the experimental cohort received PFBN treatment, while 349 patients in the control cohort were managed with InterTAN nail. Patients in all enrolled studies had a mean age > 65 years, and the classification methods for intertrochanteric femoral fractures included AO classification or Evans classification. Table 2 presents the baseline characteristics of the included studies. Regarding intervention measures, the included studies elaborated on the operational procedures of the two surgical modalities in detail, covering core technical aspects such as surgical incision localization and internal fixation implantation steps, while comprehensively documenting perioperative management strategies to ensure the reproducibility of the research methods. For outcome indicators, assessments were conducted from multiple dimensions including functional prognosis (e.g., HHS), surgical safety (e.g., intraoperative haemorrhage, complications), and rehabilitation progress (e.g., postoperative weight-bearing duration, length of hospital stay), fully covering key clinical efficacy observation points. Nevertheless, as PFBN represents an improved surgical technique developed in recent years, no randomized controlled trials focusing on the comparative analysis between PFBN and InterTAN nail have been published to date. This shortage of high-level controlled research inevitably lowers the overall quality of available clinical evidence in this field.

**Table 2:** Characteristics of the enrolled investigations

Author	Year	Studytypes	Age		Sex (Male/Female)		AO (A1.3/A2.2/A3.1/A3.3) /Evans (I/II/III/IV/V) Classification		Sample size	
			experimental	control	experimental	control	experimental	control	experimental	control
Chai [42]	2025	PS	73.19±6.96	72.15±7.24	19/27	18/32	8/15/18/5	12/11/15/12	46	50
Fu [43]	2024	RCS	76.27±4.47	80.55±7.05	10/12	7/13	6/7/6/3	13/3/2/2	22	20
Jia [40]	2023	RCS	83.28±7.85	79.90±9.60	8/17	7/13	8/7/6/4	6/5/5/4	25	20
Jin [18]	2024	RCS	73.67±5.16	73.45±5.34	9/16	14/26	3/10/10/2	1/3/19/16/1	25	40
Li [41]	2025	PS	69.30±3.82	69.99±3.25	120/80	119/81	NA	NA	200	200
Wan [35]	2024	RCS	78.00±8.80	75.30±7.00	5/11	4/15	4/9/3	1/15/3	16	19

### Risk of bias assessment

**Fig. 2:**
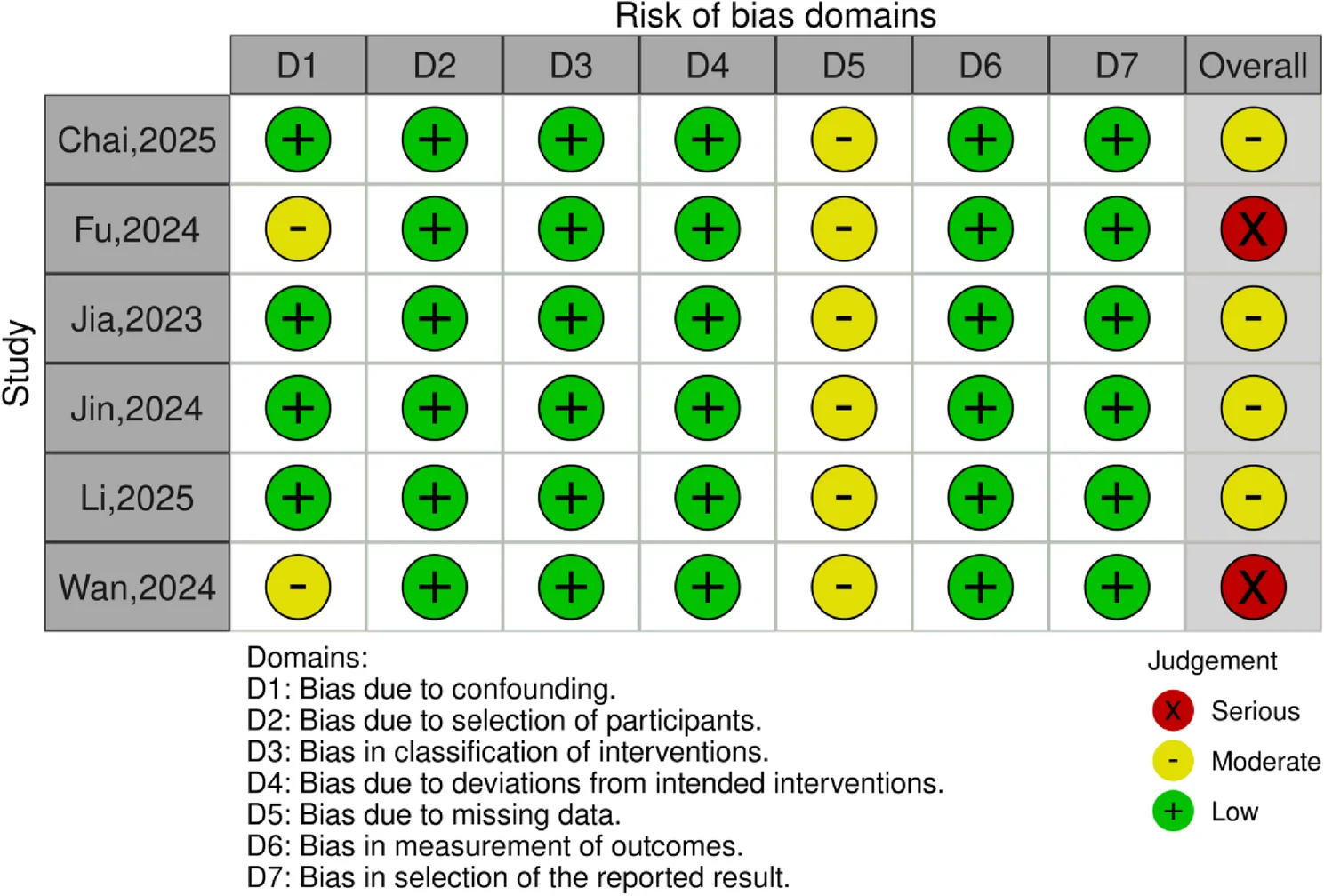
Bias risk plot

**Fig. 3:**
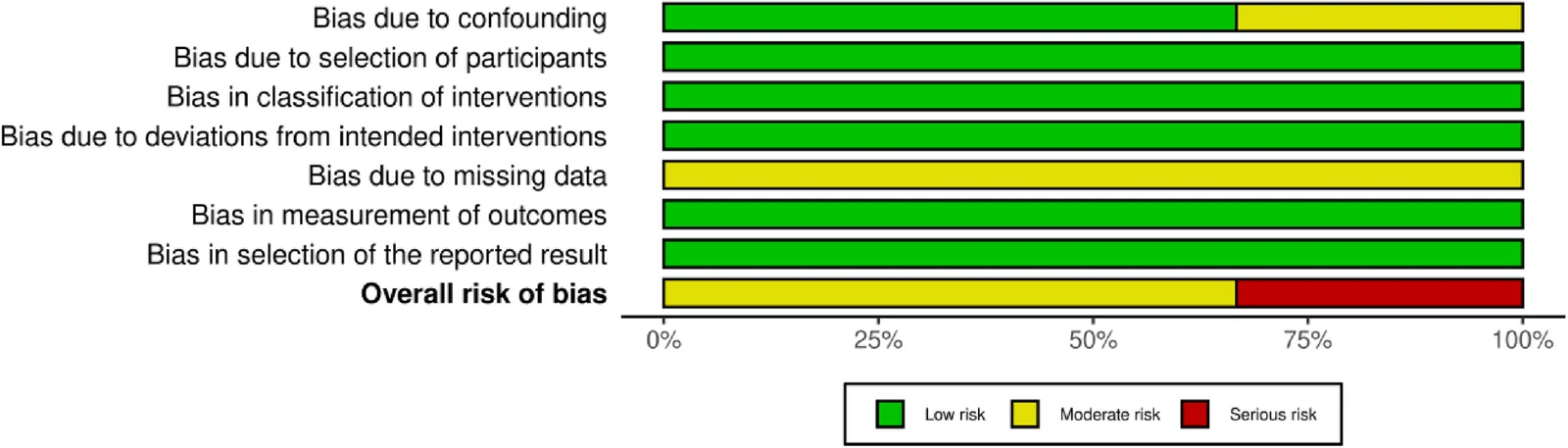
Risk of bias summary chart

The methodological quality of included studies was systematically evaluated utilizing the ROBINS-I checklist (Cochrane Risk of Bias Assessment Tool; Figs. 2 and 3). Four studies were rated as moderate risk of bias, and two at serious risk. Confounding bias and inadequate strategies for handling missing data were identified as the primary contributors to these risk ratings. Using the GRADE grading system for systematic assessment, six outcome indicators were appraised in total. Among these clinical endpoints, two were graded as moderate-certainty evidence, two as low-certainty evidence, and the remaining two as very low-certainty evidence (Supplementary Material 2).

### Meta analysis results

#### Intraoperative duration

**Fig. 4:**
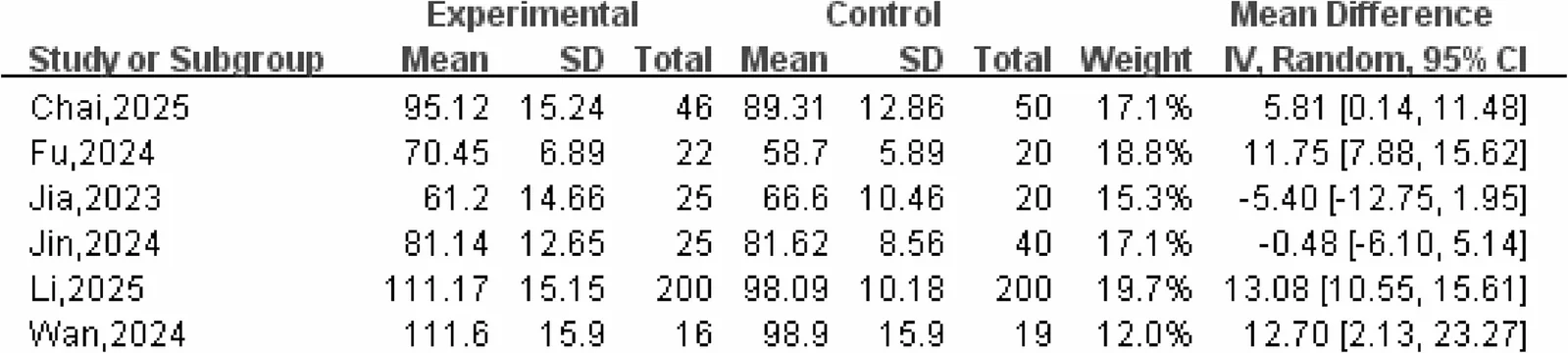
Forest plot illustrating intraoperative duration differences between the experimental cohort and the control cohort

Six included studies (*n* = 683; 334 experimental, 349 control) were analyzed for intraoperative duration. The forest plot (Fig. 4) demonstrated that the intraoperative duration in the experimental cohort was longer than that in the control cohort [MD = 6.4, 95% CI: (0.69 to 12.10), *P* = 0.03], with a heterogeneity test result of I² = 87%, indicating substantial heterogeneity. Given the inclusion of fewer than 10 studies, qualitative and quantitative assessments of publication bias were not performed, nor were subcohort analyses, meta-regression, or other heterogeneity source explorations conducted; instead, a random-effects model was directly applied for analysis. Upon further review of the included studies, such substantial heterogeneity was found to be mainly attributable to two key factors. First, none of the enrolled studies stratified patients based on fracture classification. Second, variations in surgical experience and learning curves collectively contributed to the marked between-study heterogeneity.

#### Intraoperative haemorrhage

**Fig. 5:**
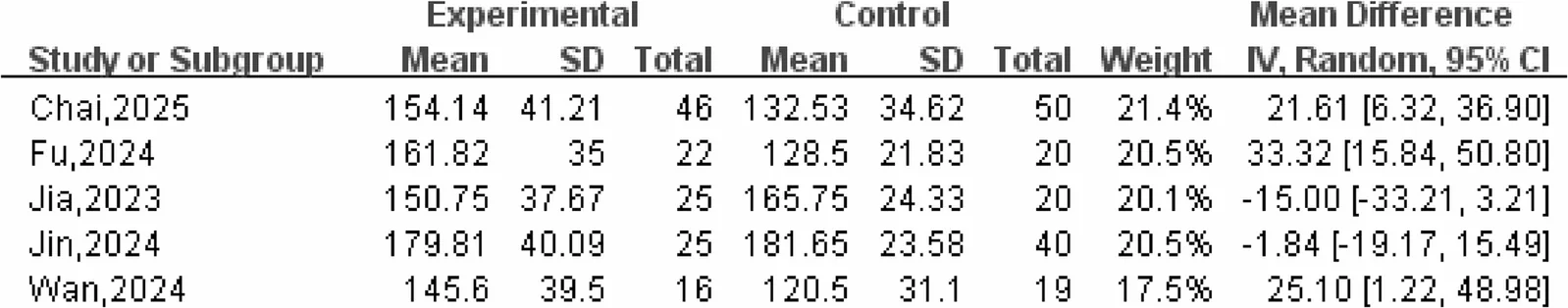
Forest plot of intraoperative haemorrhage between experimental cohort and control cohort

Five studies (n = 283; 134 experimental, 149 control) were enrolled in the meta-analysis of intraoperative haemorrhage. No statistically significant disparity between the cohorts was observed [MD = 12.44, 95% CI: (-5.34 to 30.22), P = 0.17] (Fig. 5). Substantial heterogeneity was observed (I² = 79%), therefore, a random-effects model was used. The high between-study heterogeneity may be partly explained by the lack of stratification according to fracture classification and stability, which may partly account for the high heterogeneity.

#### Length of hospital stay

**Fig. 6:**
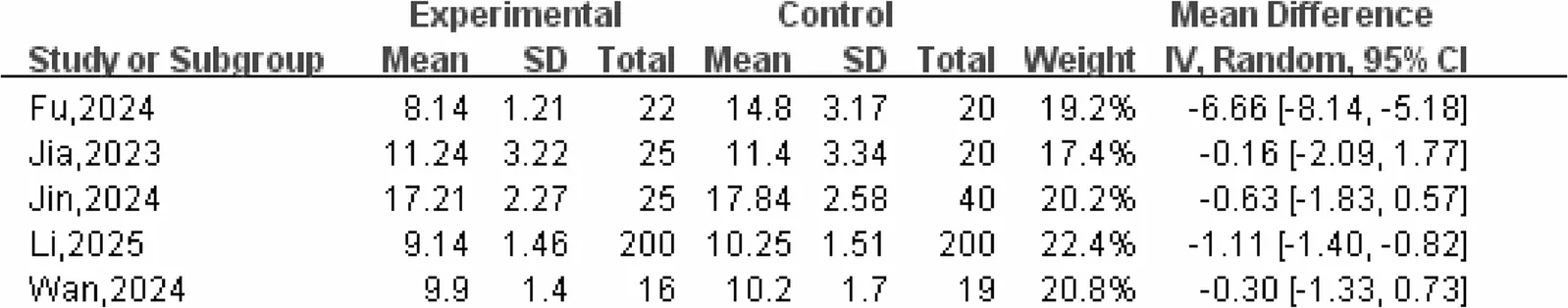
Forest plot of length hospital stay between experimental cohort and control cohort

Five studies (*n* = 587; 288 experimental, 299 control) were enrolled in the meta-analysis of length of hospital stay. Forest plot (Fig. 6) showed that the experimental cohort had a significantly shorter length of hospital stay than the control cohort [MD=-1.75, 95% CI: ( -3.44 to -0.05), *P* = 0.04]. Significant heterogeneity was identified (I² = 93%), and therefore a random-effects model was used. Analyses indicated that the substantial heterogeneity in length of hospital stay may be attributable to multiple factors, including variation in implant selection, postoperative rehabilitation protocols, family care support, and discharge criteria, driving the substantial between-study heterogeneity.

#### Postoperative weight-bearing duration

**Fig. 7:**
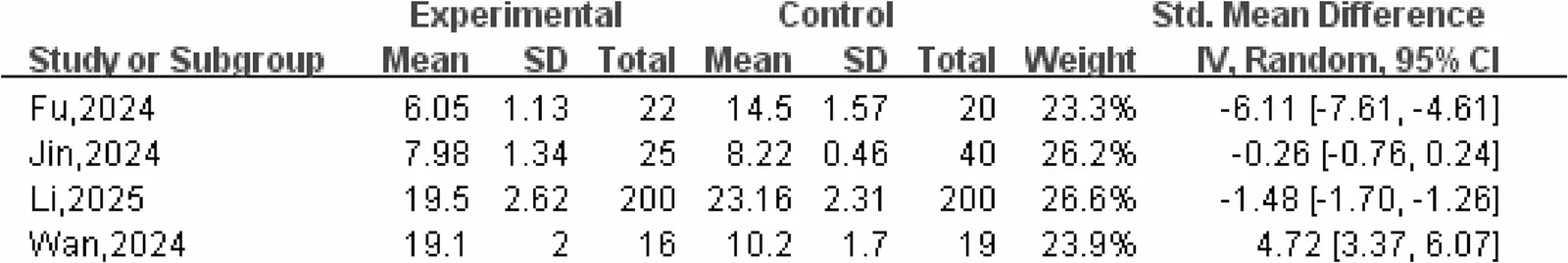
Forest plot of postoperative weight-bearing duration between experimental cohort and control cohort

Four studies (n = 542; 263 experimental, 279 control) were enrolled in the meta-analysis of postoperative weight-bearing duration. The final follow-up postoperative weight-bearing measurements were adopted for pooled analytical comparison. No significant between-cohort difference was noted [SMD = -0.76, 95% CI: (-2.81 to 1.29), P = 0.47] (Fig. 7). Substantial heterogeneity (I² = 98%) prompted direct use of a random-effects model. The lack of stratification by fracture type and stability may contribute to the high heterogeneity.

#### Harris hip score

**Fig. 8:**
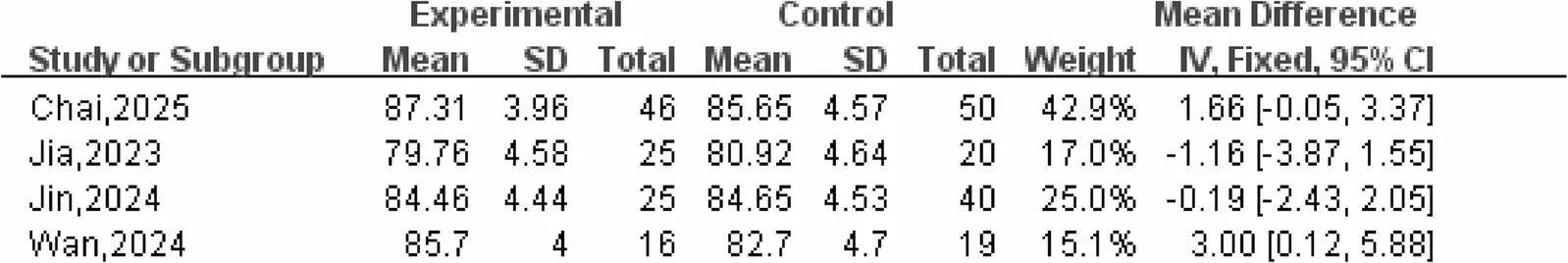
Forest plot comparing harris hip score between experimental cohort and control cohort

Four studies (*n* = 241; 112 experimental, 129 control) were included in the meta-analysis of hip joint function assessed using the HHS. The final follow-up HHS measurements were adopted for pooled analytical comparison. Forest plot (Fig. 8) indicated no statistically significant between-cohort difference in HHS [MD = 0.92, 95% CI: (-0.20 to 2.04), *P* = 0.11]. Moderate heterogeneity was noted (I² = 49%).

#### Internal fixation-related complications

**Fig. 9:**
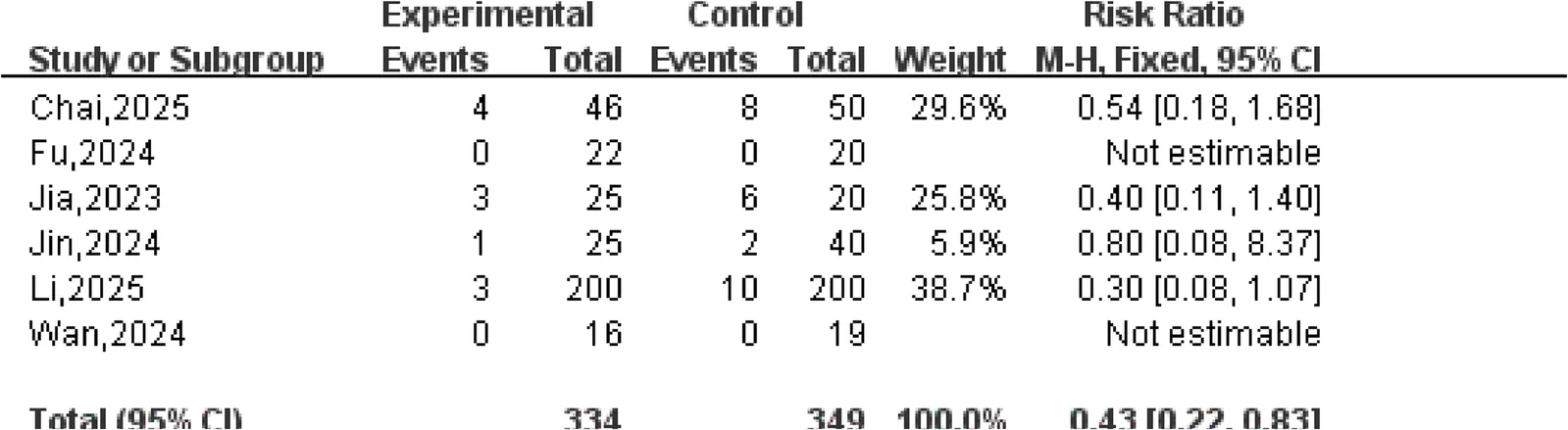
Original forest plot of internal fixation-related complications

**Fig. 10:**
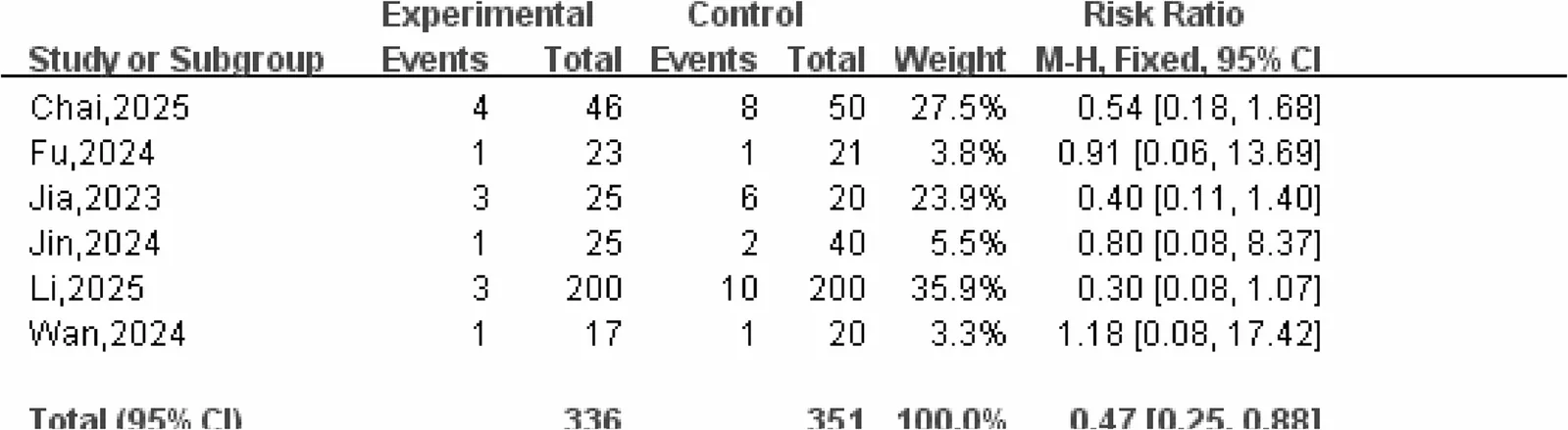
Forest plot of internal fixation-related complications after the “plus-one correction method”

Six included studies were subjected to meta-analysis using a fixed-effects model. Forest plot (Fig. 9) showed the experimental cohort had a significantly lower risk of internal fixation-related complications [RR = 0.43, 95% CI: (0.22 to 0.83), Z = 2.49, P = 0.01], with no pronounced heterogeneity (P = 0.86, I² = 0%). The included complications in this analysis encompassed screw cut-out, screw back-out, coxa vara, implant fracture and reoperation. Although these individual clinical endpoints were not regarded as interchangeable, they were all grouped as internal fixation-related complications for pooled quantitative synthesis. To address potential bias from “zero events,” a sensitivity analysis using the plus-one correction method was conducted (Fig. 10). Post-correction, the experimental cohort still had a significantly lower complication risk [RR = 0.47, 95% CI: (0.25 to 0.88), Z = 2.36, P = 0.02; I² = 0%], confirming consistent findings. Further risk difference (RD) analysis (Figure S1) revealed a 4% lower absolute complication risk in the experimental cohort [RD=-0.04, 95% CI: (-0.08 to -0.01), Z = 2.52, P = 0.01], further corroborating its superiority. In summary, across RR, RD, and corrected analyses, the experimental cohort exhibited a significantly lower risk of internal fixation-related complications, with stable findings and minimal heterogeneity.

#### Sensitivity analyses

Sensitivity analysis via sequential omission of each study revealed that after removing any single study, heterogeneity did not change substantially. Furthermore, the point estimate of the combined effect size remained within the 95% confidence interval (CI) of the overall pooled effect, indicating favorable general stability of the findings (Figure S2 - Figure S6). In conclusion, despite the presence of substantial heterogeneity in this meta-analysis, the pooled effect estimate remained relatively stable upon sensitivity analysis.

## Discussion

### Clinical efficacy analysis

Meta-analysis results indicated no distinct differences between the two groups in terms of intraoperative haemorrhage, postoperative weight-bearing duration, and Harris Hip Score (HHS). Evidence synthesized from available observational studies suggested that the proximal femoral bionic nail (PFBN) may reduce internal fixation-related adverse events. A shorter hospital stay may also theoretically reduce immobilization-related complications, although these outcomes were not directly pooled in the present meta-analysis. Meanwhile, this bionic internal fixation implant may reduce internal fixation‑related adverse events, potentially facilitating early functional recovery and shorter rehabilitation, and may lower overall healthcare resource utilization. These findings were broadly consistent with the core tenets of Enhanced Recovery After Surgery (ERAS). Nevertheless, PFBN is a recently developed modified internal fixation device, and relevant randomized controlled trials are currently unavailable, which reduces the certainty of available evidence . In addition, the total intraoperative time for PFBN was significantly prolonged compared with that of the conventional InterTAN nail. This time discrepancy was largely attributable to the steep learning curve for PFBN implantation, and this difference may decrease with accumulating surgeon experience.

### Strengths

As a novel intramedullary fixation technology modified by Chinese scholars, this study systematically summarizes the clinical evidence for PFBN, with rigorous literature retrieval reducing retrieval and selection bias as much as possible on the pooled effect size. Furthermore, we applied rigorous statistical approaches, effectively enhancing the reliability and reproducibility of our findings. This study provides preliminary observational evidence for the clinical application of PFBN in the treatment of elderly IFFs.

### Limitations

All eligible studies in the current analysis were carried out exclusively within China, which limits external generalizability of our pooled conclusions. Given the absence of published randomized controlled studies on this topic and the recent publication timeframe of included studies, the overall quality of evidence is limited. In addition, several original investigations included relatively small sample sizes and presented substantial heterogeneity, accompanied by potential risks of selection and performance bias. Furthermore, the majority of primary studies adopted a short-term follow-up design, which limits comprehensive assessment of the long-term clinical efficacy and safety of this surgical procedure.

### Implications

This systematic review and meta-analysis objectively compared the clinical efficacy of PFBN and InterTAN nail for geriatric IFFs, advancing clinical research into PFBN use. It provides preliminary evidence-based data for orthopaedic clinicians, and offers a promising yet investigational implant option for geriatric IFFs.

## Conclusion

This meta-analysis demonstrates that both PFBN and InterTAN nail achieve favorable clinical outcomes in the management of intertrochanteric femoral fractures among elderly patients. In comparison with InterTAN nail, PFBN holds the potential to shorten hospital stay and lower the incidence of internal fixation-associated complications. Nevertheless, given the inherent limitations of the included studies, all findings of the present meta-analysis warrant cautious interpretation. Meanwhile, the long-term efficacy and safety of PFBN require verification in high-quality, large-scale randomized controlled trials (RCTs) with extended follow-up.

## Supplementary Information


Supplementary Material 1.



Supplementary Material 2.



Supplementary Material 3.


## Data Availability

All data generated or analyzed during this study are included in this published article.

## Abbreviations

IFFs: Intertrochanteric femoral fractures
PFBN: Proximal femoral bionic nail
DHS: Dynamic hip screw
PFNA: Proximal femoral nail anti-rotation
PRISMA: Preferred Reporting Items for Systematic Reviews and Meta-Analyses
MeSH: Medical Subject Headings
HHS: Harris Hip Score
RR: Risk ratio
OR: Odds ratio
WMD: Weighted mean difference
SMD: Standardized mean difference
CI: Confidence intervals
GRADE: Certainty of the evidence using the Grading of Recommendations, Assessment, Development, and Evaluations
ERAS: Enhanced Recovery After Surgery
RCTs: Randomized controlled trials
