# Supplementary material for: Proximal femoral bionic nail (PFBN) versus InterTAN nail for intertrochanteric femoral fractures in elderly patients: a systematic review and meta-analysis
Source: BMC Surg. 2026 Jun 20;26:592. doi: 10.1186/s12893-026-03943-8 (PMC13536653; doi:10.1186/s12893-026-03943-8)
Supplement: Supplementary file 1 [file 12893_2026_3943_MOESM2_ESM.pdf]

Pubmed:
(((Intertrochanteric Femoral Fractures[Mesh]) OR (Fractures[Title/Abstract] OR Hip Fractures[Title/Abstract] OR Femoral Fracture[Title/Abstract] OR Proximal Femoral Fracture[Title/Abstract] OR Intertrochanteric[Title/Abstract] OR Intertrochanteric Femoral Fractures[Title/Abstract])) AND (Proximal Femoral Bionic Nail[Title/Abstract] OR PFBN[Title/Abstract])) AND (InterTAN Nail [Title/Abstract] OR InterTAN[Title/Abstract]) 12

Web of science:
((proximal femur bionic nail)) OR (PFBN)) AND (InterTAN) 12

Embase：
((proximal femur bionic nail)) OR (PFBN)) AND (InterTAN) 9

Cochrane:
((proximal femur bionic nail)) OR (PFBN)) AND (InterTAN) 1

万方(Wanfang)：
（ 题名或关键词:(PFBN) and 题名或关键词:(InterTAN) ） 7
Translations: (Title/Keywords: PFBN) AND (Title/Keywords: InterTAN)

知网(CNKI)：
（关键词：股骨近端仿生髓内钉）OR（关键词：PFBN） 11
Translations:(Keyword: Proximal femur bionic nail) OR (Keyword: PFBN)

维普(VIP)：
(题名或关键词:(PFBN) and 题名或关键词:(InterTAN)) 3
Translations: (Title/Keywords: PFBN) AND (Title/Keywords: InterTAN)
