# Supplementary material for: Proximal femoral bionic nail (PFBN) versus InterTAN nail for intertrochanteric femoral fractures in elderly patients: a systematic review and meta-analysis
Source: BMC Surg. 2026 Jun 20;26:592. doi: 10.1186/s12893-026-03943-8 (PMC13536653; doi:10.1186/s12893-026-03943-8)
Supplement: Supplementary file 2 [file 12893_2026_3943_MOESM1_ESM.docx]

Supplementary Materials for the Meta-Analysis

Figure S1 Forest Plot of Internal Fixation-Related Complications After Supplementary Risk Difference (RD) Correction.

Figure S2 Sensitivity Analysis of Intraoperative Duration.

Figure S3 Sensitivity Analysis of Intraoperative Haemorrhage.

Figure S4 Sensitivity Analysis of Length of Hospital Stay.

Figure S5 Sensitivity Analysis of Duration of Weight-Bearing.

Figure S6 Sensitivity Analysis of Harris Hip Score.
