# Supplementary material for: Proximal femoral bionic nail (PFBN) versus InterTAN nail for intertrochanteric femoral fractures in elderly patients: a systematic review and meta-analysis
Source: BMC Surg. 2026 Jun 20;26:592. doi: 10.1186/s12893-026-03943-8 (PMC13536653; doi:10.1186/s12893-026-03943-8)
Supplement: Supplementary file 3 [file 12893_2026_3943_MOESM3_ESM.docx]

**Supplementary Material 2**

****Table 1**** Certainty of the evidence using the Grading of Recommendations, Assessment, Development, and Evaluations (GRADE) assessment tool; HHS: Harris Hip Score, PFBN: proximal femoral biomic nail, InterTAN; CI: confidence interval, MD: mean difference.
